# Instability Testing for Congenital Hip Dislocation: Knee Extension Provokes Hip Dislocation

**DOI:** 10.7759/cureus.8107

**Published:** 2020-05-14

**Authors:** Panagiotis V Samelis

**Affiliations:** 1 First Orthopaedic Department, Children’s General Hospital Panagiotis & Aglaia Kyriakou, Athens, GRC; 2 Orthopaedics, Orthopaedic Research and Education Center, Attikon University Hospital, Athens, GRC

**Keywords:** congenital, hip, dislocation, barlow, ortolani, knee, extension, spica, developmental, dysplasia

## Abstract

The classic Ortolani and Barlow signs are routinely used to diagnose hip instability secondary to severe acetabular dysplasia in the newborn. However, eliciting a positive sign depends largely on the experience of the examiner and the subjective amount of manual pressure the examiner applies on the baby's hips. Furthermore, these signs do not give a clue for the selection of a maturation or immobilization device after reduction of an unstable hip: below-knee hip spica, above-knee hip spica or a Pavlik harness. The aim of this study is to describe a clinical sign that could be useful in detecting hip instability of the newborn and to decide the proper treatment in a more objective manner: knee extension provokes dislocation of the ipsilateral unstable hip.

## Introduction

Decades have passed since Marino Ortolani (1937) and Thomas Geoffrey Barlow (1962) described the homonymous tests for hip instability to early diagnose congenital hip dislocation in infants [1,2]. The Ortolani test reduces a dislocated hip (Ortolani hip, resting-dislocated hip, reducible hip), while the Barlow test dislocates an unstable hip (Barlow hip, resting-reduced hip, dislocatable hip). Both situations occur in the background of severe acetabular dysplasia. If positive, these signs alarm for further evaluation of the hip and for immediate initiation of treatment. Interestingly, in their classic manuscripts, both authors typically perform the respective manoeuvers with the knees of the infants fully flexed [1,2].

Clinical and experimental data link knee extension with ipsilateral acetabular dysplasia and hip dislocation. An ultrasonographic study in dislocated hips showed that, when the hips were flexed, ipsilateral knee extension further displaced the femoral head in the direction of the dislocation [3]. This movement of the femoral head was attributed to the increased tension of the hamstrings with knee extension [3]. Frank breech presentation with the knees in extension is a serious predisposing factor for congenital hip dislocation and hip dysplasia [4]. Experimental data have shown that the previously healthy hips of rabbits dislocate, if the knee joints are splinted in extension [5]. Selective cutting of the hamstrings spared the respective hip from dislocation in spite of permanent ipsilateral knee extension [5].

These data suggest that knee extension may harm normal acetabular development by transmitting tension through the hamstrings to the acetabular cartilage anlage. These muscles (biceps femoris, semimembranous, semitendinous) span both the hip and knee joints.

The aim of this study is to describe the clinical effect of knee extension on the ipsilateral unstable or dislocated hip of the newborn. Is this manoeuver useful to detect hip instability and to decide treatment options for an unstable or a dislocated hip?

## Case presentation

**Figure 1:**
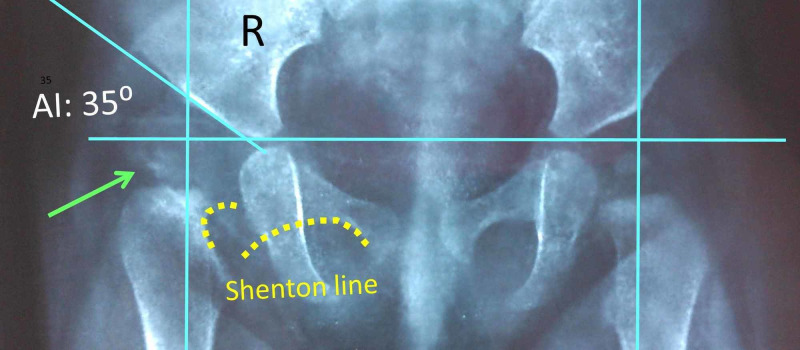
Anteroposterior pelvis view of the patient. Radiologic signs of hip instability and acetabular dysplasia of the right hip are evident: disruption of Shenton line (dotted line), an acetabular index of 35 degrees and irregular ossification of the laterally displaced right femoral head (arrow), compared to the normal contralateral side. R: right, AI: acetabular index

**Figure 2:**
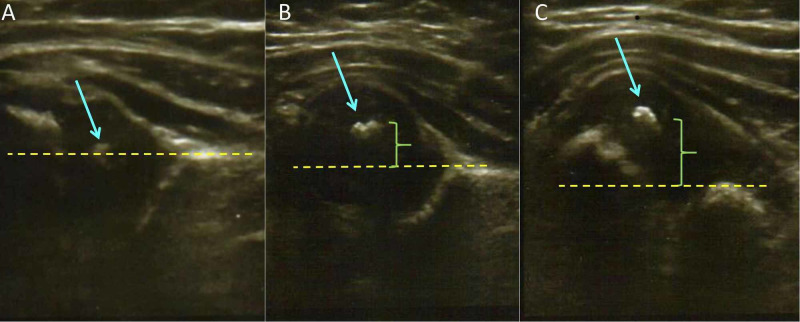
Dynamic ultrasound examination of the right hip of the patient (A) Reduced hip: the ossific nucleus of the femoral head (arrow) is at the same level with the innominate bone (dotted line). (B) Subluxed hip: the ossific nucleus (arrow) moves away (bracket) from the level of the innominate bone (dotted line). (C) Dislocated hip: the ossific nucleus (arrow) of the femoral head is displaced laterally and proximally (bracket) relative to the acetabular rim (dotted line).

A term born, second born, non-breech presentation, caesarean section, seven-month-old girl was referred to the hospital with a diagnosis of a congenitally dislocated right hip. She had a negative family history for hip disease. Limited abduction of the right hip compared to the healthy contralateral side, asymmetrical skin folds of the right thigh and a positive Galleazi sign (shorter right limb) indicated a resting dislocated (Ortolani) right hip. Anteroposterior X-rays of the pelvis and ultrasound examination of the right hip confirmed the diagnosis (Figures 1, 2).

Clinical signs of instability were positive. The right hip was reduced using the Ortolani manoeuver and was redislocated when the hip was abducted less than 45 degrees. Restricted abduction of the right hip compared to the contralateral side, indicating adductor muscle contraction, was evident. Due to the relatively delayed diagnosis, the patient was placed in longitudinal skin traction for one week, in order to stretch soft tissues around the hip joint and to facilitate closed reduction.

The knee-extension-hip-dislocation manoeuver

After one week in traction, the patient was examined under general anaesthesia. The right hip reduced with maximal abduction (about 80 degrees) using the Ortolani manoeuver. From this position of maximal abduction (and while in 90 degrees of flexion), the right hip was gradually brought to the minimally abducted position, where the femoral head was still stably seated in the acetabulum (about 45 degrees of abduction). In the clinical setting, this is the position the surgeon would prefer to immobilize the hip in order to treat the patient.

The question is, is the hip joint really stable with hip abduction at 45 degrees and hip flexion at 90 degrees, irrelevant of the position of the knee? And what would be the preferred treatment? A below-knee hip spica, an above-knee hip spica or a Pavlik harness?

**Figure 3:**
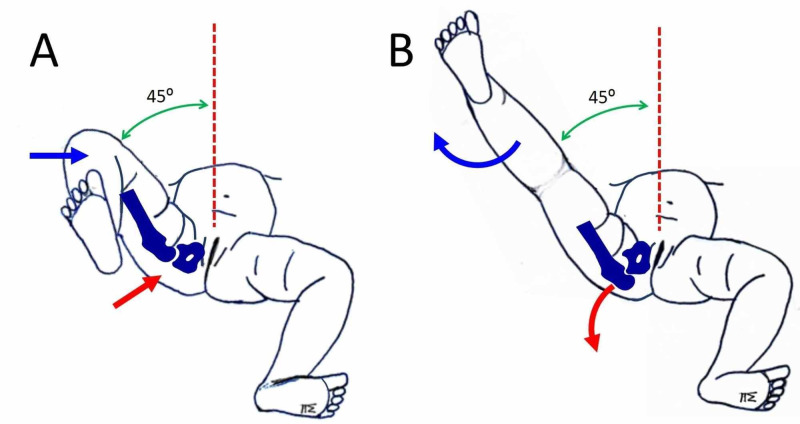
The knee-extension-hip-dislocation manoeuver (A) The right hip is reduced following the Ortolani manoeuver (red arrow). The ipsilateral knee is fully flexed (blue arrow). The right hip is gradually brought to the midline (dotted line). The minimal amount of abduction with the hip reduced is manifested. In this patient, the right hip re-dislocated with abduction less than 45 degrees (green arrow). (B) With the right hip reduced at 45 degrees of abduction (green arrow), gradual extension of the ipsilateral knee starts (blue arrow). At some point, resistance to further knee extension is felt. Further attempt to extend the right knee dislocates the right hip (red arrow).

At this point, knee extension started. At 60-80 degrees of knee flexion, some resistance was felt, while the examiner was applying force on the tibia. After a few more attempts to extend the right knee, the right hip re-dislocated (Figure 3).

**Figure 4:**
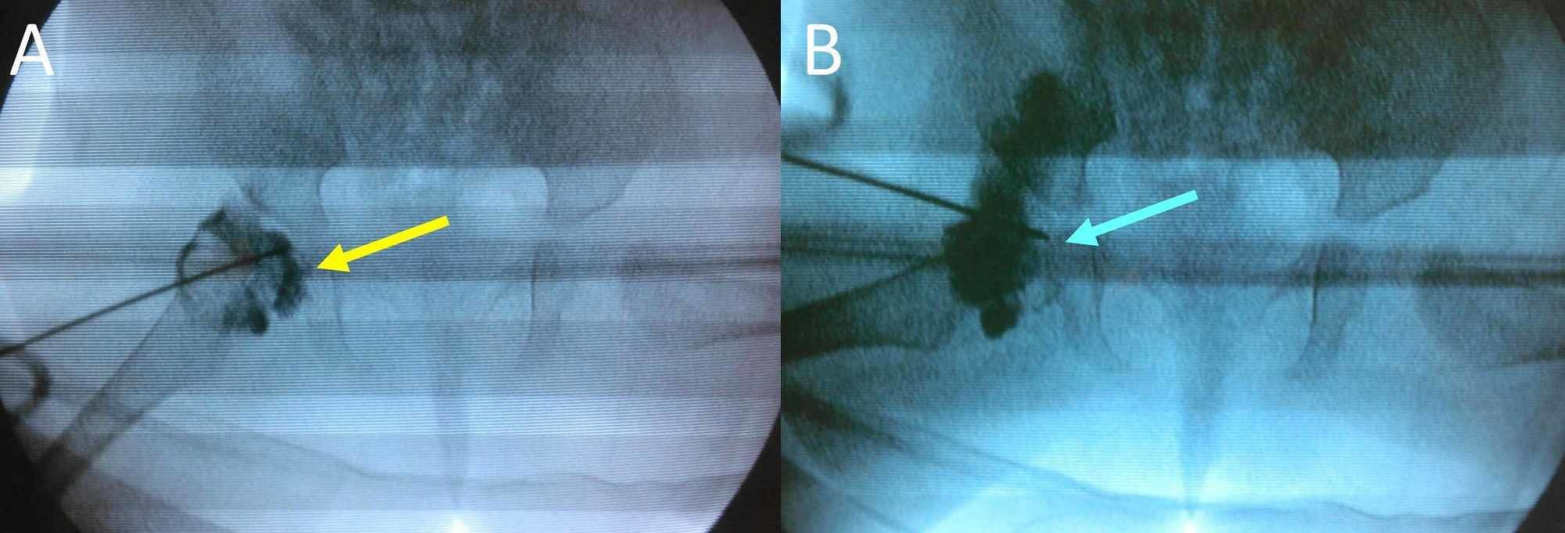
Arthrogram of the right hip of the patient (A) Dislocated right hip. The radiopaque fluid (yellow arrow) accumulates between the femoral head and the acetabular floor. (B) Anatomical closed reduction of the femoral head into the acetabulum with hip abduction is confirmed by the complete disappearance of the radiopaque fluid between the femoral head and the acetabular floor (blue arrow). Open reduction is not necessary.

An arthrogram of the right hip followed. Anatomical reduction of the femoral head in the acetabulum (femoral head in contact with the acetabular floor) was confirmed with hip flexion at 90-100 degrees and hip abduction at 45 degrees (Figure 4).

**Figure 5:**
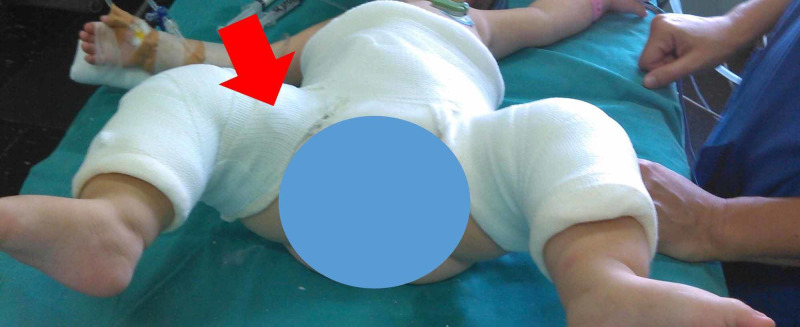
Immobilization of the hips of the patient by means of a below-knee hip spica The right hip (red arrow) was placed in the position of stability determined arthrographically and clinically. Extending the spica below the knee eliminates the adverse effect of knee extension on the dysplastic hip.

Percutaneous adductor tenotomy was performed in order to release tension of the contracted adductors of the right hip. As expected, adductor tenotomy facilitated hip abduction. However, hip dislocation with knee extension was unaffected by adductor tenotomy, indicating involvement only of the hamstrings on the knee-extension-hip-dislocation manoeuver. Immobilization with a hip spica in the arthrographically determined the position of stable reduction (hip flexion 100 degrees, hip abduction 45-50 degrees) followed. Extension of the hip spica below the knee, with the knee in 90 degrees of flexion, was decided to avoid the adverse effect of knee extension on hip stability (Figure 5).

**Figure 6:**
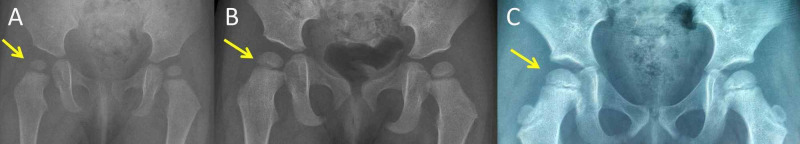
Follow-up anteroposterior pelvis x-rays of the patient Gradual resolution of acetabular dysplasia of the right hip (arrows) is observed at the age of 12 months (A), 24 months (B) and four years (C).

The patient was followed in regular intervals. After a few weeks, the nucleus of ossification of the affected hip appeared. The hip spica was removed after four months according to the clinical examination and the radiographic appearance of the hip. The development of the right hip improved significantly. Complete resolution of acetabular dysplasia was manifested at the age of four years (Figure 6).

## Discussion

The harmful effect of knee extension on normal hip development has been noticed since decades, but this observation has not been useful in the clinical setting [3-5]. Thus, diagnosis and treatment of an unstable or dislocated hip never took into account the position of the ipsilateral knee.

Further attention should be paid on the effect of knee extension on the developing hip of the neonate and the infant. Depending on the duration of dislocation of a resting dislocated hip, the muscles of the thigh may be contracted [2]. After hip reduction using the Ortolani manoeuver, the tension of the hamstrings, which span both the hip and the knee joints, is determined by the motion of the ipsilateral knee [3]. Knee extension stretches the hamstrings and, subsequently, promotes re-dislocation of the femoral head.

Knee extension has never been part of the clinical examination for hip instability of the newborn. Eliciting the classic signs of hip instability, as described by Ortolani and Barlow, depends largely on the experience of the examiner and the subjective manual pressure that the examiner applies on the baby's hips [1,2,6]. On the contrary, hip dislocation with ipsilateral knee extension is an objective manoeuver, since it depends on the passive movement of the patients’ knee throughout a predetermined normal range of motion of the knee joint.

Furthermore, ipsilateral knee extension has never been considered in selecting between a maturation or an immobilization device for the treatment of an unstable hip: Pavlik harness, above-knee hip spica or below-knee hip spica. Ideally, the preferred treatment after hip reduction should balance between proper abduction of the hip (avoid avascular necrosis) and flexion of the ipsilateral knee (avoid tensioning the hamstrings).

A Pavlik harness or an above-knee hip spica does not prevent knee extension. A Pavlik harness does not even prevent hip adduction. Selecting these devices for the treatment of an unstable hip, especially a resting-dislocated (Ortolani) hip, may impose some risk on acetabular development and treatment outcome. Novais et al. reported that Pavlik harness treatment failed in 27% of Ortolani-positive hips, compared with 8% of stable dysplastic hips and 5% of Barlow-positive hips [7]. High failure rates of the Pavlik harness in dislocated hips have been reported by other authors as well [8].

It is proposed that a Pavlik harness or an above-knee hip spica should be reserved for hips with a negative knee-extension-hip-dislocation test. Such hips are stable dysplastic hips, Barlow hips (resting reduced) and, probably, very early diagnosed Ortolani hips (resting dislocated). A positive knee-extension-hip-dislocation test indicates contracted hamstrings and mandates a below-knee hip spica with the knee flexed to avoid tensioning the hamstrings, either by involuntary movements of the baby or during care-taking of the baby by the parents.

## Conclusions

Clinical and experimental evidence confirm the effect of knee extension on hip stability during the last weeks of gestation and after birth, by means of increasing the tension of the hamstrings. Opposite to the Barlow and Ortolani tests, the knee-extension-hip-dislocation test depends not on the subjective force applied by the examiner, but also on the completion of the normal range of motion of the knee of the newborn and infant. This test may be helpful for the primary detection of hip instability; however, a large case series is needed to provide this information. At the moment, the knee-extension-hip-dislocation test is useful to assess the position of stability after hip reduction and to decide whether a Pavlik harness, an above-knee hip spica or a below-knee hip spica should be selected for the treatment of developmental dysplasia of the hip in neonates and infants. After reduction of a resting-dislocated (Ortolani) hip, a below-knee hip spica with the knee in 90 degrees of flexion may be a safe choice to prevent the harmful effect of ipsilateral knee extension on the dysplastic acetabulum.
